# Food Choice and Nutrition: A Social Psychological Perspective

**DOI:** 10.3390/nu7105424

**Published:** 2015-10-21

**Authors:** Sarah J. Hardcastle, Cecilie Thøgersen-Ntoumani, Nikos L.D. Chatzisarantis

**Affiliations:** Health Psychology and Behavioural Medicine Research Group, School of Psychology and Speech Pathology, Faculty of Health Sciences, Curtin University, GPO Box U1987, Perth, WA 6845, Australia; c.thogersen@curtin.edu.au (C.T.-N.); nikos.chatzisarantis@curtin.edu.au (N.L.D.C.)

**Keywords:** food choice, healthy eating, nutrition, social psychology

## Abstract

In this Special Issue, entitled “Food choice and Nutrition: A Social Psychological Perspective”, three broad themes have been identified: (1) social and environmental influences on food choice; (2) psychological influences on eating behaviour; and (3) eating behaviour profiling. The studies that addressed the social and environmental influences indicated that further research would do well to promote positive food choices rather than reduce negative food choices; promote the reading and interpretation of food labels and find ways to effectively market healthy food choices through accessibility, availability and presentation. The studies on psychological influences found that intentions, perceived behavioural control, and confidence were predictors of healthy eating. Given the importance of psychological factors, such as perceived behavioural control and self-efficacy, healthy eating interventions should reduce barriers to healthy eating and foster perceptions of confidence to consume a healthy diet. The final theme focused on the clustering of individuals according to eating behaviour. Some “types” of individuals reported more frequent consumption of fast foods, ready meals or convenience meals or greater levels of disinhibition and less control over food cravings. Intervention designs which make use of multi-level strategies as advocated by the Ecological Model of Behaviour change that proposes multi-level (combining psychological, social and environmental) strategies are likely to be more effective in reaching and engaging individuals susceptible to unhealthy eating habits than interventions operating on a single level.

The Special Issue on the social psychological issues related to food choice and nutrition has attracted a wide variety of papers from around the world and across population groups. Three broad themes were identified through the papers: (1) social and environmental influences on food choice; (2) psychological influences on eating behaviour; and (3) eating behaviour profiling.

Six papers focused on the social and environmental influences on food choice. Deliens *et al.* [1] surveyed university students and found that those exposed to modelling influences (*i.e*., having family and friends who rarely consume soft drinks), stricter family rules, greater perceived behavioural control and confidence were less likely to consume soft/energy drinks [1]. Tanja *et al.* [2] surveyed adolescents to explore the relationship between eating competence and food choices. They found that greater eating competence was associated with greater meal frequency, a higher intake of fruits and vegetables and more health-promoting family eating patterns [2]. These findings align with previous research. For example, fewer household rules controlling food and eating [3,4] and free availability of energy dense foods in shops and at home [5] positively influence obesity in children and adolescents. In addition, other researchers have found modelling to be an important influence whereby parents who consumed a high intake of fruit and vegetables were more likely to have children who also exhibit high fruit and vegetable intake [6].

Other social-environmental papers published in this issue focused on attention to food labels [7] and environmental-based interventions to encourage low calorie snacks [8] or plant-based foods [9]. Miller and colleagues [7] set up a mock shopping task and monitored eye movements to assess attention to nutritional information on food labels in US adults. Miller *et al.* [7] found that those who paid more attention to food labels were more likely to consume a healthy diet. Miller *et al.*’s [7] study is one of the first to demonstrate that food label use is related to diet quality. Bos and colleagues [8] conducted an online survey across three time points to explore acceptance of intervention strategies for low-calorie snack choices that vary in the impact they have on consumers’ freedom of choice (providing information, guiding choice through (dis)incentives and restricting choice) in an adult Dutch population. Bos *et al*. [8], found that perceptions of personal and societal effectiveness and fairness positively influenced acceptance of interventions for low-calorie snack choices. Further, encouraging low calorie snacks rather than discouraging high calorie choices was better received. Finally, Ensaff and colleagues [10] conducted focus groups with adolescents to explore attitudes towards plant-based foods and factors influencing food choices. Ensaff *et al*. [10], found that taste, appearance, personal food history, habits and familiarity were important influences on food choice. Such findings are important because if individuals are not exposed to vegetable based meals at home, they are less likely to choose plant-based foods elsewhere. Barriers to healthy food choices including taste and convenience will be revisited later in relation to eating behaviour profiling. Future research and interventions would do well to find ways of introducing healthy plant-based foods to individuals and demonstrating that such foods can be tasty. Ensaff and colleagues also examined the effect of a simple intervention designed to improve the accessibility, availability and presentation of healthy food items (*i.e*., whole fruit, fruit salad, vegetarian daily specials) in a school canteen. Their results showed that the intervention was effective in facilitating subsequent selection of more healthy food choices among secondary school students. Taken together, these studies suggest that further research would do well to promote positive food choices rather than reduce negative food choices; promote the reading and interpretation of food labels and find ways to effectively market healthy food choices through food architecture models.

The second main theme in this set of papers is centred around the psychological influences on eating behaviour. Perceived behavioural control (the perceived ease or difficulty in performing a behavior) and confidence were found to statistically predict eating behaviour in several studies involving university students [1,11] and young adults [12]. In relation to vegetable intake, the cross-sectional study by Menozzi and colleagues [11] found that intentions and perceived behavioural control explained 68% of vegetable consumption in Italian students. In a similar vein, using a cross sectional design Deliens *et al.* [1] found that University students with higher perceived behavioural control, confidence and subjective norm were less likely to consume soft drinks. Low levels of confidence concerning the satiating capacity of food were also associated with higher energy consumption among young adults in the study by Schiöth and colleagues [12]. Finally, Dimmock and colleagues [13] suggested that quality of motivation, as depicted in Self-determination theory is likely to influence cognitive processes such that those with controlled types of motivation will be susceptible to post-exercise consumption of pleasurable but unhealthy foods. Behaviour change theories, including the Theory of Planned Behaviour [14], Social Cognitive Theory [15] and Self-Determination Theory [16,17] appear useful to understand the processes underpinning eating behaviour. Interventions designed to improve eating behaviour could be based on such theories in the future with a view to ascertain cause and effects.

The final theme identified was a focus on eating behaviour profiling or the clustering of individuals according to eating behaviours. Two papers used approaches to identify typologies of individuals [18,19]. Dalton and colleagues’ [18] crossover study on female participants recruited from a University campus found a distinct low satiety phenotype characterised by high resting metabolic rate, greater levels of disinhibition who also self-reported lower control over food cravings. Those individuals characterised by the low satiety phenotype also consumed more energy. Along similar lines, Sarmugam and Worsley [19] identified three types of individuals in relation to eating behaviors: “impulsive involved”, “uninvolved”, and “rational, health conscious”. The first two types reported more frequent consumption of fast foods, ready meals or convenience meals and salted snacks compared to the rational health conscious types. Sarmugam and Worsley proposed several environmental strategies (supermarkets) to target and engage the two types of individuals susceptible to unhealthy eating habits. These included low-budget initiatives to appeal to the uninvolved, and in-store marketing cues or prompts (*i.e*., end of aisle displays) to influence the impulsive involved group. They also found that impulsive involved individuals relied heavily on ready-made sauces and mixes which may indicate a lack of cooking skills. As such, healthy eating interventions may do well to promote the use of healthier processed foods such as canned and frozen vegetables and beans in cooking rather than focusing on cooking from scratch using fresh ingredients. Other research found that a main outcome of a cookery skills intervention was that participants learnt how to make healthy meals from scratch that were both tasty and time efficient [4]. The importance of food being “tasty” has been emphasised by Vidgen and Gallegos [20]. It may be that the acquisition of cooking skills may change the ways in which foods are perceived.

In conclusion, both socio-psychological and environmental strategies appear effective in changing eating behaviour and associated outcomes. It would be interesting in future research to employ intervention designs which make use of multi-level strategies as advocated by the Ecological Model of Behaviour Change [21], which proposes that multi-level (combing psychological, social and environmental) strategies are likely to be more effective than interventions operating on a single level. Environmental approaches, such as food architecture interventions, may be a promising way to prompt healthy food choices, and in doing so reach those individuals that tend to be more impulsive purchasers. Further, given the findings reported in this issue and elsewhere on modelling and household rules governing food consumption, family based interventions may also be important. Such interventions may focus on ways to prepare and cook quick tasty meals such that barriers to healthy eating may be reduced (*i.e*., barriers concerning time, cost and taste) and confidence to prepare health meals be enhanced. Additionally, further work is required on food labels, both in terms of who responds to them and how people make sense of them. Finally, given the importance of psychological factors such as perceived behavioural control and self-efficacy, healthy eating interventions should reduce barriers to healthy eating and foster perceptions of confidence to consume a healthy diet. Health behaviour change theories, including those outlined above, may be usefully applied to foster such confidence.
